# Case Report: D-bifunctional protein deficiency caused by novel compound heterozygote *HSD17B4* variants in a neonate in China

**DOI:** 10.3389/fgene.2025.1631767

**Published:** 2025-11-06

**Authors:** Hui Liu, Gaojie Liu, Lianjun Gao, Hui Wang, Yizhong Wang, Hongfang Ding

**Affiliations:** 1 Department of Pediatrics, Shengli Oil Field Central Hospital, Dongying, Shandong, China; 2 Department of Gastroenterology, Hepatology, and Nutrition, Shanghai Children’s Hospital, School of Medicine, Shanghai Jiao Tong University, Shanghai, China; 3 Gut Microbiota and Metabolic Research Center, Institute of Pediatric Infection, Immunity and Critical Care Medicine, School of Medicine, Shanghai Jiao Tong University, Shanghai, China

**Keywords:** D-BPD, *HSD17B4*, VLCFAs, neonate, seizure

## Abstract

**Background:**

D-bifunctional protein deficiency (D-BPD) is a rare fatal autosomal recessive peroxisomal disorder caused by biallelic pathogenic mutations in the hydroxysteroid 17-beta dehydrogenase 4 (*HSD17B4*) gene; it is characterized by hypotonia, seizures, and facial dysmorphisms during the neonatal period.

**Case presentation:**

In this report, we describe a female neonate from China who was diagnosed with D-BPD. The patient presented with neonatal asphyxia, hypotonia, weak reflexes, and feeding difficulty after birth. Seizures occurred on the fifth day of life and were initially treated with phenobarbital. However, the seizures reoccurred and became more difficult to control because of their increased frequency, duration, and anticonvulsive drug resistance. Whole-genome sequencing (WGS) revealed novel compound heterozygous mutations c.1145G>A(p.Gly382Asp)/c.1193C>G(p.Ser398*) in exon 13 of the *HSD17B4* gene, which was confirmed by parental Sanger sequencing. Neither variant has been reported previously. Very-long-chain fatty acid (VLCFA) testing revealed markedly elevated levels of hexacosanoic acid (C26:0), tetracosanoic acid/docosanoic acid (C24:0/C22:0), and C26:0/C22:0. The patient was managed with formula nasogastric feeding and antiepileptic therapy. At 7 months of age, she demonstrated severe psychomotor retardation, inability to grasp and manipulate objects, no language development, hearing loss, and poor visual response.

**Conclusion:**

We described the incidence of D-BPD in a Chinese neonate caused by novel biallelic pathogenic variants in *HSD17B4*, which expands its mutational spectrum.

## Introduction

D-bifunctional protein deficiency (D-BPD, MIM 261515), first described by Watkins et al. (1989), is a rare autosomal recessive peroxisomal disorder with an estimated prevalence of 1:30,000 to 1:100,000 newborns (Ferdinandusse et al., 2006). D-BPD patients are typically characterized by hypotonia, seizures, facial dysmorphisms, psychomotor delay, blindness, and deafness during the neonatal period, which leads to severe developmental delay and early death within the first 2 years of life in most cases (Ognean et al., 2024). D-BPD is caused by biallelic pathogenic mutations in the hydroxysteroid 17-beta dehydrogenase 4 (*HSD17B4*) gene located on chromosome 5q23.1, which encodes a D-bifunctional protein (D-BP) of 736 amino acids (Moller et al., 2001). D-BP is a peroxisomal enzyme with three active domains: 3-hydroxyacyl-CoA dehydrogenase (N-terminal), 2-enoyl-CoA hydratase (central), and a C-terminal sterol carrier protein 2-like domain. D-BP catalyzes the hydration (second step) and dehydrogenation (third step) reactions in bile acid synthesis and the peroxisomal β-oxidation of fatty acids, including very-long-chain fatty acids (VLCFAs) and branched fatty acids (Hashimoto, 2000). Although the majority of D-BPD patients survive less than 2 years, some can achieve a longer life. Ferdinandusse et al. (2006) explored the correlation between the enzyme level and clinical outcome by dividing the D-BPD patients based on their survival. Higher residual activity for C26:0 β-oxidation and lower C26:0 levels in skin fibroblast cultures, as well as lower plasma C26:0 levels, were positively associated with longer survival.

D-BPDs are classified into three major types on the basis of their activity deficiency in the three functional domains: type I—both dehydrogenase and hydratase deficiency; type II—isolated hydratase deficiency; type III—only dehydrogenase deficiency (Ferdinandusse et al., 2006). The three types share similar clinical features but differ in terms of severity. Recently, additional types of IV and V, similar to Perrault syndrome (PRLTS) (OMIM #233400), with less severe clinical manifestations than types I, II, and III have been described (McMillan et al., 2012; Pierce et al., 2010). The diagnosis of D-BPD involves several steps, including clinical evaluation, biochemical testing, and genetic analysis (Ognean et al., 2024). This multifaceted diagnostic process is challenging for practitioners, as the clinical features, disease course, and imaging findings are not unique compared to other peroxisomal disorders (e.g., Zellweger syndrome) (FitzPatrick, 1996). Furthermore, biochemical markers, including VLCFA, bile acids, and phytanic acid, may not be consistently elevated in D-BPD-affected individuals (Landau et al., 2020; Soorani-Lunsing et al., 2005). Although skin fibroblast cultures for enzyme studies allow for testing of enzymatic activity, they are generally unavailable and take time. Thus, genetic testing is the last critical step for the definitive diagnosis of D-BPD.

To date, only a few D-BPD patients have been reported in the Chinese population (Chen et al., 2021; Hsu et al., 2024; Yang et al., 2021). In this study, we present a case from China of neonatal-onset D-BPD caused by the novel compound heterozygote mutations c.1145G>A(p.Gly382Asp)/c.1193C>G(p.Ser398*) in the *HSD17B4* gene. The clinical features and disease course of the patient are described in the study.

## Case presentation

A female newborn was delivered by Cesarean section because of fetal distress during labor after 40 weeks of gestation in a local hospital, with a birth weight of 2.57 kg (<3rd percentile) and head circumference of 31 cm. The Apgar score was not known. The baby girl was born to a non-consanguineous couple of Chinese Han ethnicity; the mother was 34 years old, and the father was 33. The girl was the first child for both parents, and there was no history of seizures/epilepsy, developmental delay, or early childhood deaths in the family. The amniotic fluid was sticky and contaminated with meconium (grade III), and the umbilical cord was fecally contaminated and tightly wrapped once around the neck. The newborn girl had no spontaneous breathing, no heartbeat, and pale skin. Positive pressure ventilation (PPV) with a laryngeal mask and epinephrine infusion were performed immediately after birth. The heart rate was approximately 109 beats per minute, and blood oxygen saturation was 100% after 7 min of epinephrine injection; the patient was then transferred to the neonatal intensive care unit (NICU) of a superior-level hospital (our hospital) under positive pressure ventilation 45 min after birth.

**FIGURE 1:**
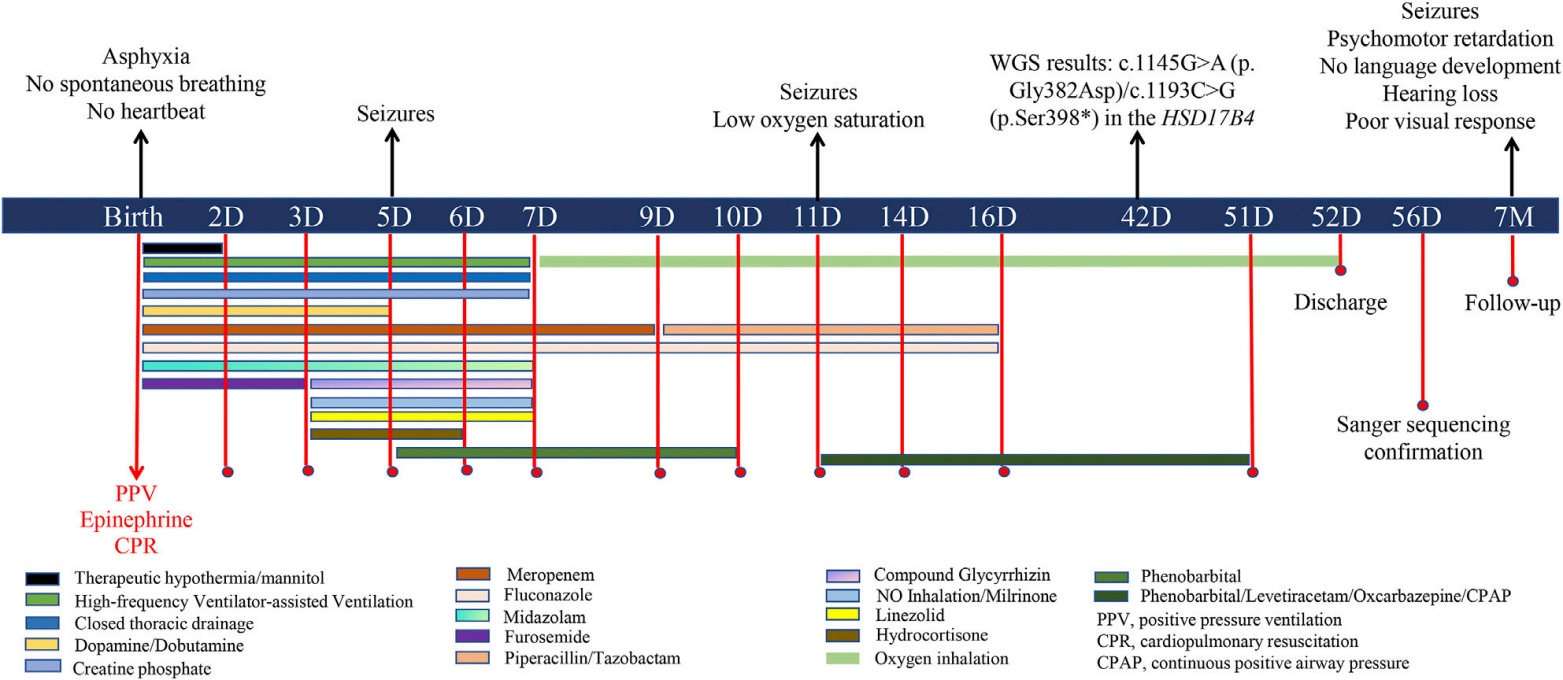
Diagram outlining clinical events, diagnostics, and treatments of the patient.

On admission, physical examination revealed a body temperature of 35.5 °C, no spontaneous breathing, no heartbeat, and a blood pressure of 40/20 mmHg. The skin appeared pale and ecchymotic, without rashes, dehydration or edema. No significant facial dysmorphism was observed. Both pupils were dilated, and the pupillary light reflex was absent. The spine and limbs were normal, but the muscle tone of the limbs was weak. The baby did not have spontaneous movement, and the rooting reflex, sucking reflex, grasping reflex, and Moro reflex were absent. Tracheal intubation was reperformed under PPV, and external chest compression was carried out for cardiopulmonary resuscitation (CPR). Vital signs were absent for 10 min, and the heart rate returned to 90 beats per minute after infusion of epinephrine, with a blood oxygen saturation of approximately 85%. Afterward, the patient was supported with tracheal intubation and high-frequency ventilator-assisted ventilation for respiratory failure. Blood gas analysis revealed pH 6.79 (normal range: 7.35–7.45), PCO2 81.32 mmHg (reference range: 35–45 mmHg), PO2 26.52 mmHg (reference range: 80–100 mmHg), HCO3 8.30 mmol/L (reference range: 21.4–27.4 mmol/L), base excess −23.39 mmol/L (reference range: −3–3 mmol/L), and lactic acid 16.91 mol/L (normal range: 0.5–1.6 mmol/L), indicating severe metabolic acidosis and high lactic acidosis. A blood routine test showed elevated white blood cells (WBCs, 38.39^∗^10^∧^9/L, reference range: 5–25^∗^10^∧^9/L) and platelets (311^∗^10^∧^9/L reference range: 100–260^∗^10^∧^9/L) with low levels of red blood cells (RBC, 3.90^∗^10^∧^9/L, reference range: 10–12^∗^10^∧^9/L) and hemoglobin (135 g/L, reference range: 140–220 g/L). Other laboratory tests showed significantly increased alanine aminotransferase (ALT, 430 U/L, reference range: 13–45 U/L), aspartate aminotransferase (AST, 1316 U/L, reference range: 25–76 U/L), serum neuron-specific enolase (NSE, 136 ng/mL, reference range: 0–16.3 ng/mL), creatine kinase (CK, 9335 U, reference range: 40–200 U), CKMB (195.80 ng/mL, reference range: 0–5 ng/mL), and interleukin 6 (IL-6, 3,430 pg/mL, reference range: 0–6 pg/mL); C-reactive protein (CRP) level was normal (3.3 mg/mL, reference range: 0–6). Cranial ultrasound showed no obvious dilation in the ventricles and cisterns. X-ray revealed pneumothorax and compression of lung tissue on the right side. As summarized in Figure 1, the patient was treated with therapeutic hypothermia for suspected hypoxic–ischemic encephalopathy (HIE) and prophylactic antibiotics (e.g., meropenem, linezolid, piperacillin, tazobactam, and fluconazole) to prevent infection and early-onset sepsis (EOS). Intermittent furosemide diuresis was applied to reduce brain cell edema, and mannitol to reduce intracranial pressure. Glycyrrhizin was given for liver protection and lowering transaminase levels. Dopamine, dobutamine, and creatine phosphate were used for cardiogenic shock, neonatal hypotension, and myocardial damage. Closed thoracic drainage on the right side was performed for pneumothorax and compression of lung tissue. NO inhalation and milrinone were applied for persistent pulmonary hypertension of the newborn (PPHN). In addition, hydrocortisone was used for anti-inflammation and for increasing sensitivity to vasoactive drugs. The patient received nasogastric feeding because of feeding difficulties.

On the fifth day of life, frequent twitching of the eyelids and mouth corners, lateral nystagmus, and convulsions were noted and confirmed by electroencephalography (EEG) as seizures. The patient’s epileptic seizures progressed from focal to generalized seizures. During drowsiness, there were five to seven episodes of spasms, each characterized by limb lifting at 10–20 s intervals, sometimes accompanied by 7–8 s tremors of both legs. EGG showed a broad high-amplitude slow-wave complex with a low-amplitude 15–20 Hz fast-wave rhythm lasting approximately 1 s and sharp-wave rhythms in the bilateral central–parietal and mid-temporal regions for 7–8 s. Phenobarbital (5 mg/kg/day) was administered to control the seizures for 5 days. A series of blood, cerebrospinal fluid (CSF), and sputum cultures were all negative. Brain magnetic resonance imaging (MRI) performed at 10 days of life was normal. However, the seizures reoccurred on the 11th day of life and were accompanied by low oxygen saturation. The patient was supported by continuous positive airway pressure (CPAP), and intravenous phenobarbital (5 mg/kg/day) and oral levetiracetam were provided to treat the seizures. The patient presented with recurrent seizures several times a day despite adjustments in anticonvulsant drugs (phenobarbital, levetiracetam, and oxcarbazepine) and dosage. HIE and EOS as the etiology of seizures were thus excluded based on the normal MRI imaging and negative blood, CSF, and sputum cultures.

**FIGURE 2:**
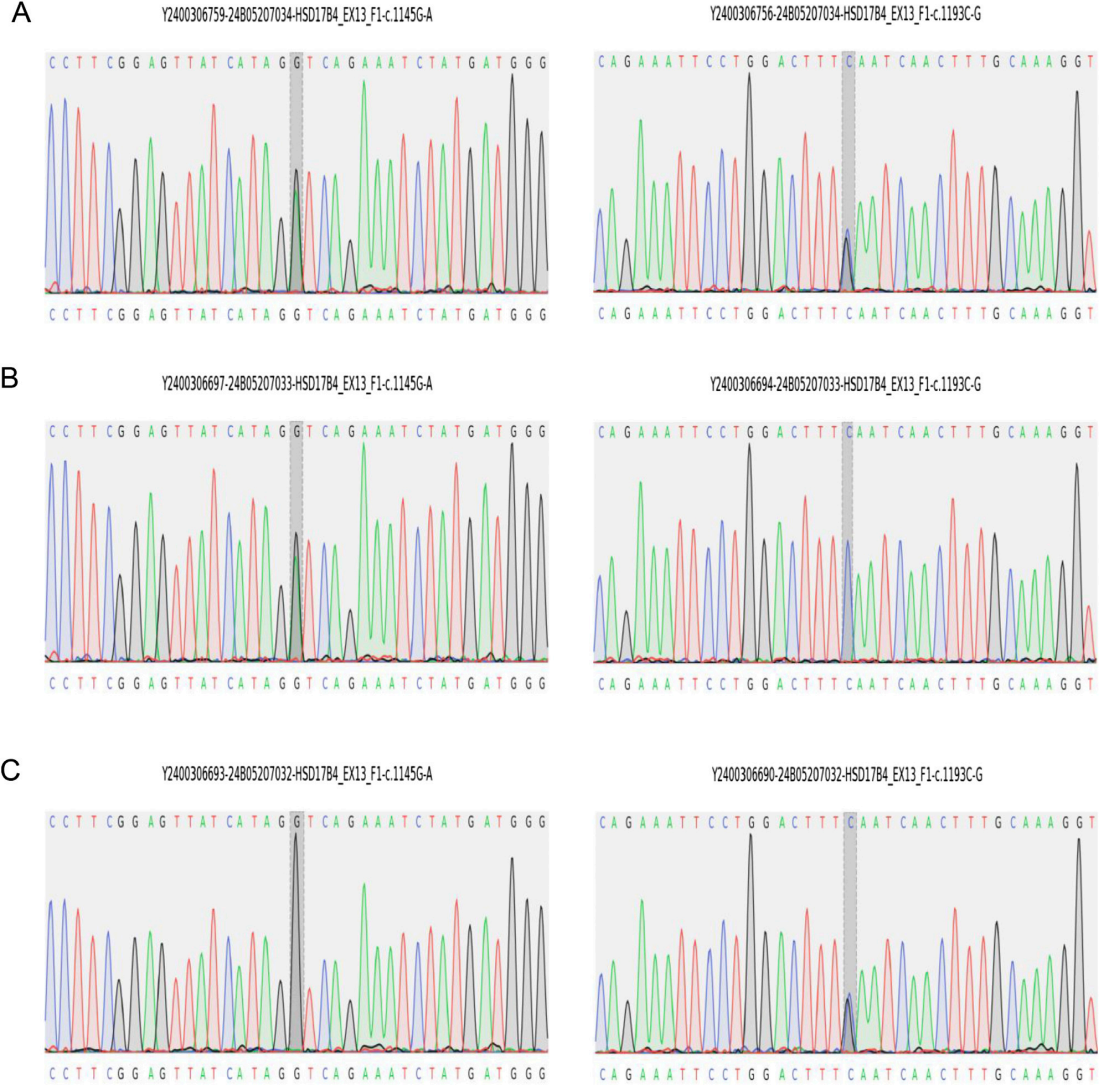
Sanger sequencing validation of *HSD17B4* variants identified by whole-genome sequencing in the family. **(A)** Proband carries both variants of c.1145G>A(p.Gly382Asp) and c.1193C>G(p.Ser398*). **(B)** The father carries c.1145G>A(p.Gly382Asp). **(C)** The mother carries c.1193C>G(p.Ser398*).

At age 14 days, the patient was suspected of having genetic metabolic disorders on the grounds of hypotonia and intractable epilepsy. To evaluate the genetic etiology of neonatal seizures, whole-genome sequencing (WGS) was performed using genomic DNA extracted from the peripheral blood of the patient. The WGS results on the 42nd day of life of the patient revealed novel compound heterozygous mutations c.1145G>A(p.Gly382Asp)/c.1193C>G(p.Ser398*) in exon 13 of the HSD17B4 gene, which were confirmed by Sanger sequencing (Figure 2A) on the 56th day of life. The c.1145G>A variant results in the amino acid substitution of glycine for aspartic acid at codon 382 (p.Gly382Asp). The c.1193C>G(p.Ser398*) variant leads to a premature termination codon at codon 398, and the aberrant transcript is likely degraded by nonsense-mediated mRNA decay (NMD). Neither variant has been reported by the 1000 Genomes Project, gnomAD, or the literature. The c.1145G>A(p.Gly382Asp) variant is defined as a variant of uncertain clinical significance (PM2 + PM3), and the c.1193C>G(p.Ser398*) variant is defined as likely pathogenic (PVS1 + PM2) according to American College of Medical Genetics and Genomics (ACMG) guidelines (Miller et al., 2021). The variant c.1145G>A(p.Gly382Asp) was predicted to be pathogenic by multiple bioinformatic algorithms. It is absent from gnomAD exome database and ExAC database and was predicted to be damaging by SIFT, Alpha-Missense, and Mutation Taster (Table 1). Genotyping of the unaffected parents revealed the variant c.1145G>A(p.Gly382Asp) in the father (Figure 2B) and the c.1193C>G(p.Ser398*) mutation in the mother (Figure 2C). VLCFA testing revealed markedly elevated levels of hexacosanoic acid (C26:0, 9.55 nmol/mL; reference range: ≤ 1.30 nmol/mL), tetracosanoic acid/docosanoic acid (C24:0/C22:0, 2.07; reference range: ≤1.39), and C26:0/C22:0 (0.238; reference range: ≤0.023), which confirmed the diagnosis of D-BPD.

**TABLE 1:** *In silico* analyses of the pathogenic impact of variant c.1145G>A (p.Gly382Asp) in *HSD17B4.*

Gene	Variant	Position in chromosome (hg19)	gnomAD	ExAC	Alpha-missense	SIFT (score)	Mutation taster
*HSD17B4*	c.1145G>A (p.Gly382Asp)	chr5:118835184	-	-	0.8799	Deleterious (0.02)	Disease causing

**FIGURE 3:**
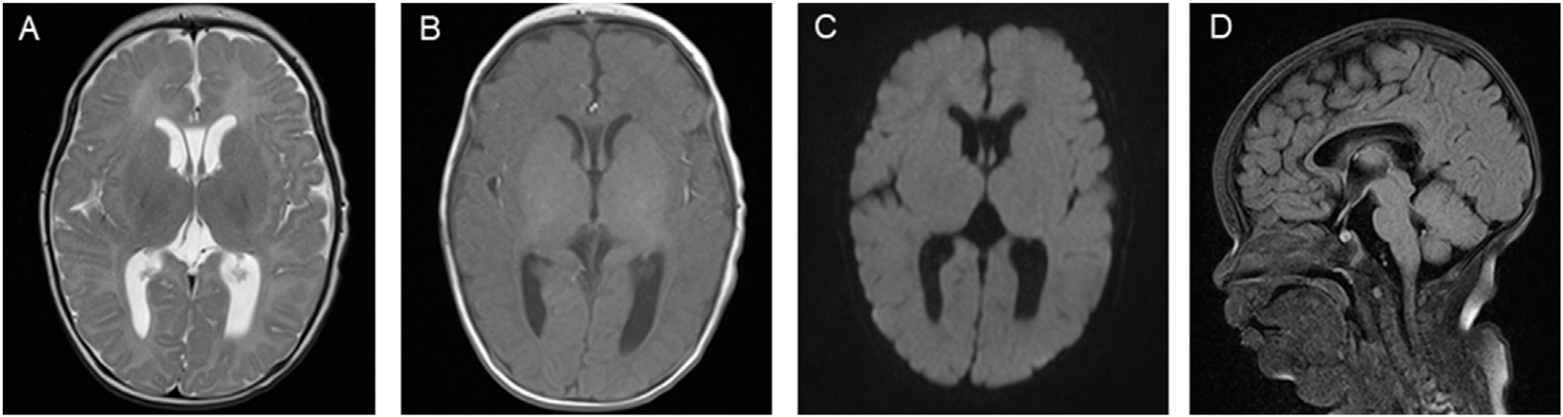
Brain magnetic resonance imaging (MRI) images on the patient’s 51st day of life. **(A)** T2-weighted imaging (T2WI) showing symmetrical bilateral cerebral hemispheres with normal gray and white matter signal intensities. **(B)** T1-weighted imaging (T1WI) showing high signal intensity in the posterior limb of the internal capsule and the cavum septum pellucidum. **(C)** Diffusion-weighted imaging (DWI) showing no obvious abnormal signal. **(D)** Fluid-attenuated inversion recovery (FLAIR) sequence showing the corpus callosum genu and body with normal morphology.

These results led the patient to be diagnosed with D-BPD type II caused by the novel compound heterozygous mutation c.1145G>A (p. Gly382Asp)/c.1193C>G (p.Ser398*) in exon 13 of the *HSD17B4* gene. Before discharge, a repeated MRI was performed at 51 days of admission (Figure 3). This showed symmetrical bilateral cerebral hemispheres with normal gray and white matter signal intensities (Figure 3A). T1-weighted images (T1WI) revealed increased signal intensity in the posterior limb of the internal capsule, and the cavum septum pellucidum was present (Figure 3B). Diffusion-weighted imaging (DWI) showed no obvious abnormal signal (Figure 3C), and a fluid-attenuated inversion recovery (FLAIR) sequence showed that the corpus callosum genu and body were in normal morphology (Figure 3D). On the 52nd day of life, the patient was discharged with normal laboratory test results of blood routine, coagulation function, blood glucose, blood ammonia, blood lactic acid, blood lipids, electrolytes, liver function, and thyroid function after a series of symptomatic and supportive treatments, including antiepileptic drug therapy, oxygen inhalation, and nutrition support. However, the patient’s hypotonia and convulsive seizures were persistent, and she was prescribed anticonvulsive drugs to control the seizures and was supported with nasogastric feeding at home. The patient was closely followed-up and readmitted to the hospital multiple times for severe seizures and recurrent respiratory tract infections. At the time of writing, she is 7 months old, and physical examination reveals a severe delay in development. She presents with severe psychomotor retardation, inability to grasp and manipulate objects, no language development, hearing loss, and poor visual response (no follow-up vision). Eye examinations could not be completed because the patient was unable to cooperate. The infant demonstrates continuous feeding difficulty and is supported by nasogastric feeding. Phenobarbital (5 mg/kg, qd), levetiracetam (25 mg/kg, bid), and oxcarbazepine (12 mg/kg, bid) are used to treat the recurrent seizures. We have offered genetic counseling to the parents and strongly recommend a prenatal diagnosis for the next pregnancy. However, they do not yet plan to have a second baby.

## Discussion

D-BPD is a single-enzyme disorder caused by peroxisomal β-oxidation defects and has a high burden of morbidity and early mortality (Ferdinandusse et al., 2006). The typical clinical features of D-BPD are neonatal onset hypotonia, seizures, facial dysmorphism, and severe psychomotor delay (Ferdinandusse et al., 2006; Kasula et al., 2023; Yahia et al., 2025). A comprehensive review by Ferdinandusse et al. (2006) revealed that most patients with D-BPD present with neonatal hypotonia (98%) and seizures (93%). The patient described in this study presented with severe complications during birth, including a lack of spontaneous breathing, no heartbeat, neonatal asphyxia, hypotonia, and weak reflexes after birth. Severe complications that occurred during birth may have affected the neurological development of the patient. Although D-BPD can lead to severe clinical phenotypes, acute perinatal hypoxic–ischemic injury may further aggravate the patient’s brain injury. To the best of our knowledge, such severe birth complications in D-BPD patients have rarely been reported in the literature. A few severely affected D-BPD patients were reported to exhibit mild to moderate asphyxia-like symptoms at birth, but patients who presented with cardiac arrest and required prolonged resuscitation were rare (Ognean et al., 2024).

Seizures were observed on the fifth day of life and were confirmed by EEG in our patient, although MRI findings were normal. The majority of reported severely affected D-BPD patients showed varying degrees of abnormalities on early neuroimaging (such as MRI), including changes in basal ganglia signals, brainstem/cerebellar atrophy, delayed myelination or ventricular enlargement (Ognean et al., 2024). However, not all severe DBP patients exhibit obvious structural abnormalities in the early stage of the disease. A few cases in which normal brain MR images were obtained during the neonatal period have been reported, and nerve dysfunction may be the result of functional or metabolic disorders rather than structural damage to the brain (Chen et al., 2021; Hsu et al., 2024). The patient was initially treated with phenobarbital for convulsions. However, the seizures reoccurred on the 11th day of life and were accompanied by low oxygen saturation. The seizures became more difficult to control because of their increased frequency, duration, and anticonvulsive drug resistance. Neonatal-onset D-BPD cases have been reported in several previous studies; for example, Kasula et al. (2023) reported three D-BPD cases in which clinical signs appeared during the neonatal period, and Yahia et al. (2025) reported a case with neonatal-onset seizures and hypotonia due to D-BPD. Although most D-BPD patients show cranial and facial dysmorphisms, our patient did not present obvious facial abnormalities. Recently, Ognean et al. (2024) reported a case of D-BPD without facial dysmorphism. In addition, multisystemic manifestations of D-BPD, including retina, adrenal gland, hearing, and skeleton defects, have been reported (Bae et al., 2020; Chen et al., 2021; Incecik and Mungan, 2019; Mehtala et al., 2013). Our patient failed the hearing test and lacked a pupillary light reflex at birth, and at 7 months of age our patient was confirmed to have poor visual response and hearing loss.

D-BP, encoded by the *HSD17B4* gene, plays a critical role in the β-oxidation of fatty acids (Moller et al., 2001). The *HSD17B4* gene consists of 24 exons, with exons 1–12 coding for the 3-hydroxyacyl-CoA dehydrogenase domain, exons 12–21 coding for the 2-enoyl-CoA hydratase domain, and exons 21–24 coding for the sterol carrier protein 2-like domain (Jiang et al., 1996). Biallelic pathogenic variants of the *HSD17B4* gene affect D-BP function and activity, leading to the accumulation of fatty acids that are detrimental to the development of the central nervous system and are responsible for autosomal recessive D-BPD (Moller et al., 2001). On the basis of the site of functional domain deficiency, D-BPDs are classified into three major types. To date, more than 110 pathogenic variants in the *HSD17B4* gene have been recorded in the Human Gene Mutation Database (HGMD) (Ognean et al., 2024), but only a few D-BPD cases have been reported in China. Chen et al. (2021) described a case of neonatal-onset D-BPD caused by the novel *HSD17B*4 compound heterozygous mutations c.972+1G>T/c.727T>A (p.W243R). In addition, several case reports revealed compound heterozygous mutations c. 686-2A>T/c. 1171G>C, c. 101C>T (p.Ala34Val)/c. 1448_1460del (p.Ala483Aspfs*37), and c112+1G>A/c.394C>T (p.R132W) in the *HSD17B*4 gene, which are responsible for most D-BPD cases in China (Yang et al., 2021). The limited number of reported D-BPD patients within the Chinese population may be related to the low awareness of this rare disease. The majority of D-BPD patients in China may die before genetic diagnosis. In this report, WGS revealed novel compound heterozygous mutations c.1145G>A(p.Gly382Asp)/c.1193C>G(p.Ser398*) in exon 13 of the *HSD17B4* gene in our patient, which were respectively inherited from her mother and father. Neither variant has been reported previously; c.1145G>A (p.Gly382Asp) is defined as a variant of uncertain clinical significance, and c.1193C>G (p.Ser398*) is defined as likely pathogenic, according to ACMG guidelines. Although variant c.1145G>A (p.Gly382Asp) is defined as a variant of uncertain clinical significance according to ACMG guidelines, it was predicted to be pathogenic by multiple bioinformatic algorithms. It is absent from the gnomAD exome and ExAC databases. Furthermore, the variant was predicted to be damaging by SIFT, Alpha-Missense, and Mutation Taster. Nonsense mutations are considered to be a type of harmful variant in genetic disorders. The novel c.1193C>G(p.Ser398*) variant is a nonsense mutation that results in a premature termination codon within the coding region of *HSD17B4* at codon 398. The impact of the c.1193C>G(p.Ser398*) on the patient may lead to an aberrant transcript and result in a shortened polypeptide of *HSD17B4*. The aberrant transcript is likely degraded by NMD, and protein translation stops prematurely at codon 398, resulting in no functional full-length HSD17B4 protein being produced. Thus, our patient is predicted to have type II D-BPD. The clinical manifestations of patients with type II and III D-BPD were reported to be milder than those of patients with type I D-BPD. Although previous studies have shown that some patients with type II and III D-BPD have normal levels of VLCFAs (Landau et al., 2020; Matsukawa et al., 2017; Soorani-Lunsing et al., 2005; Yamamoto et al., 2021), our patient had markedly elevated C26:0 levels and C24:0/C22:0 and C26:0/C22:0 ratios.

The differential diagnosis of D-BPD from HIE and EOS causing neonatal seizures is complex due to the overlapping neurological, metabolic, and dysmorphic features. The seizures caused by HIE typically occur within 6–12 h post-birth, accompanied by a history of fetal distress and response to the hypothermia treatment (Douglas-Escobar and Weiss, 2015). The MRI findings include diffuse edema, basal ganglia lesions, or watershed infarcts in HIE neonates (Douglas-Escobar and Weiss, 2015). Furthermore, elevated serum NSE and S100 calcium-binding protein beta (S100B) may serve as biomarkers of seizures in HIE (Tefr Faridova et al., 2023). Our patient was suspected of having HIE due to fetal distress, neonatal asphyxia, hypotonia, and elevated serum NSE. However, the cranial ultrasound and MRI findings were normal, and seizures occurred in the fifth day of life. Seizures in EOS are associated with systemic inflammatory response and neurotoxicity caused by intrauterine or placental infection (e.g., Group B *Streptococcus*) (Car et al., 2022). The onset and remission of the seizures in EOS are roughly parallel to the outcome of the infection indicators (Andersen et al., 2025). Anti-infection treatments are normally effective for controlling seizures in EOS patients. The seizures occurred later post-birth in our patient, and there was no improvement in the frequency and form of epileptic seizures after a series of treatments, including therapeutic hypothermia, antiepileptic drugs, and anti-infection treatments, suggesting a metabolic encephalopathy. Therefore, HIE and EOS as the etiology of seizures was excluded in our patient.

Currently, there are no effective treatments for D-BPD, and most affected individuals die before 2 years of age because of severe psychomotor delay and uncontrolled seizures (Ferdinandusse et al., 2006). Rare patients with less severe manifestations may survive longer with gross motor delay and neurosensorial deficits. Soorani-Lunsing et al. (2005) reported a relatively mild case of D-BPD in an 8-year-old girl with normal VLCFA levels. Farkas et al. (2019), Khan et al. (2010), and Landau et al. (2020) reported that an 8-year-old boy, a 6-year-old girl, and an 11.5-year-old girl with D-BPD presented with leukodystrophy. Furthermore, Yamamoto et al. (2021) described a 6-year-old boy with type III D-BPD who had progressive white matter dystrophy. Interestingly, a 6-month-old boy with D-BPD caused by a homozygous *HSD17B4* c.1041T>A, p.(Tyr347Ter) nonsense variant was treated with the readthrough agent PTC124 (ataluren) for 2 years, which led to a decrease in the C26:0 level (Hsu et al., 2024). The boy demonstrated improved swallowing and progressive motor and speech development without further seizures, highlighting the potential of nonsense readthrough therapy for D-BPD (Hsu et al., 2024). However, whether normal or corrected VLCFA levels are associated with the clinical outcome of D-BPD patients is still controversial.

## Conclusion

This study described a D-BPD in a neonate from China caused by the novel compound heterozygous mutations c.1145G>A (p.Gly382Asp)/c.1193C>G (p.Ser398*) in exon 13 of the *HSD17B4* gene. Pediatricians should raise awareness of this rare hereditary disease, and genetic testing is important for the timely diagnosis of newborns with neonatal-onset hypotonia and seizures of unknown etiology. Prenatal genetic counselling is important for reducing families’ recurrent risk of D-BPD.

## Data Availability

The datasets presented in this study can be found in online repositories. The names of the repository/repositories and accession number(s) can be found in the article/supplementary material.
